# Polybrominated Diphenyl Ethers (PBDEs) and Bioaccumulative Hydroxylated PBDE Metabolites in Young Humans from Managua, Nicaragua

**DOI:** 10.1289/ehp.10713

**Published:** 2007-11-21

**Authors:** Maria Athanasiadou, Steven N. Cuadra, Göran Marsh, Åke Bergman, Kristina Jakobsson

**Affiliations:** 1 Department of Environmental Chemistry, Stockholm University, Stockholm, Sweden; 2 Facultad de Ciencias Médicas, Universidad Nacional Autónoma de Nicaragua–Managua (UNAN–Managua), Managua, Nicaragua; 3 Department of Occupational and Environmental Medicine, Lund University Hospital, Lund, Sweden

**Keywords:** brominated flame retardants, BFRs, children, fish consumption, human exposure, inhalation, metabolism, OH-PBDEs, waste disposal site

## Abstract

**Objective:**

Our aim was to investigate exposure to polybrominated diphenyl ethers (PBDEs) in a young urban population in a developing country, with focus on potentially highly exposed children working informally as scrap scavengers at a large municipal waste disposal site. We also set out to investigate whether hydroxylated metabolites, which not hitherto have been found retained in humans, could be detected.

**Methods:**

We assessed PBDEs in pooled serum samples obtained in 2002 from children 11–15 years of age, working and sometimes also living at the municipal waste disposal site in Managua, and in nonworking urban children. The influence of fish consumption was evaluated in the children and in groups of women 15–44 years of age who differed markedly in their fish consumption. Hydroxylated PBDEs were assessed as their methoxylated derivates. The chemical analyses were performed by gas chromatography/mass spectrometry, using authentic reference substances.

**Results:**

The children living and working at the waste disposal site showed very high levels of medium brominated diphenyl ethers. The levels observed in the referent children were comparable to contemporary observations in the United States. The exposure pattern was consistent with dust being the dominating source. The children with the highest PBDE levels also had the highest levels of hydroxylated metabolites.

**Conclusions:**

Unexpectedly, very high levels of PBDEs were found in children from an urban area in a developing country. Also, for the first time, hydroxylated PBDE metabolites were found to bioaccumulate in human serum.

Polybrominated diphenyl ethers (PBDEs) have been increasingly used as additive brominated flame retardants (BFRs) ever since the 1970s (Alaee et al. 2003; Bergman 2005; World Health Organization 1997) up to the point that two of the commercial BFR products—pentaBDE and octaBDE—were banned or withdrawn from the market, as was the case for these products within the European Union (Cox and Efthymiou 2003) and in North America (Great Lakes Flame Retardants 2005). PBDEs have been and are still used in textiles and in flexible polyurethane foams, as well as in electric appliances and electronic devices. Although used textiles and foams are discarded directly without any restrictions, electronic waste may either be discarded or recycled for valuable metals (Cui and Forssberg 2003). Uncontrolled discharges of material containing BFRs may lead to unintentionally high environmental exposure to these chemicals.

Humans are exposed to PBDEs via ingestion and inhalation. A large number of PBDE congeners have been reported to be present in food as reported by, for example, Schecter et al. (2006), with seafood being an important source. PBDEs are present in ambient air both in industrialized regions (Butt et al. 2004; ter Schure et al. 2004;) and in the Arctic (de Wit et al. 2006) as well as in household dust (Schecter et al. 2005a; Stapleton et al. 2005; Wu et al. 2007). Thus the dominant PBDE exposure routes differ from those of traditional persistent organic pollutants (POPs), such as polychlorinated biphenyls (PCBs). This difference appears to be attributable to numerous indoor sources for the former, but much less for the PCBs.

Most data on human PBDE exposure originate in Europe and in North America, as reviewed a few years ago (Gill et al. 2004; Hites 2004; Sjödin et al. 2003). Several more recent publications on PBDEs in humans have appeared from the United States (Bradman et al. 2007; Fischer et al. 2006; Lunder and Sharp 2004; Morland et al. 2005; Schecter et al. 2005b; She et al. 2007) and from Europe, including two-time trend studies (Covaci and Voorspoels 2005; Fängström et al. 2005, 2008; Furst 2006; Harrad et al. 2004; Ingelido et al. 2007; Jaraczewska et al. 2006; Schuhmacher et al. 2007; Thomas et al. 2006; Thomsen et al. 2007; Thuresson et al. 2005). Several reports on human PBDE exposure outside of both Europe and the United States have also been published recently. Such data have been obtained from China (Bi et al. 2006), Taiwan (Chao et al. 2007), Korea (Kim et al. 2005; Lee et al. 2007), Japan (Eslami et al. 2006; Inoue et al. 2006), Asia in general (Sudaryanto et al. 2005), Australia (Toms et al. 2007), New Zealand (Harrad and Porter 2007), and the Republic of Buryatia (Russia) (Tsydenova et al. 2007). It is clear that the PBDE contamination in North America is higher than in Europe and other parts of the world. Still, it is notable to observe concentrations of BDE-47 in the range of 2.8–9.6 ng/g fat, and a median of the sum of six individual PBDEs (∑_6_PBDEs) = 10.2 ng/g fat in Australian human milk (Toms et al. 2007), and individuals with clearly elevated levels in New Zealand, with ∑_6_PBDE concentrations in blood serum of 2.8–20.1 ng/g fat (Harrad and Porter 2007). The ∑_5_PBDE concentrations in Canada of 2.4–22 ng/g mother’s milk fat (Hites 2004) and up to 38 ng/g plasma fat in humans in Mexico (Lopez et al. 2004) indicate possible similarities to the United States (Petreas et al. 2003) in the use and distribution of PBDEs in these neighboring countries. Information from Latin America is still lacking, except for one report from Mexico (Lopez et al. 2004). There are no data from Africa, except one small breast milk study (Darnerud et al. 2006), and rarely data from most parts of Asia.

Data on exposure levels in children are extremely scarce. Notably, the few studies published on PBDE exposure indicate higher concentrations in children than in adults (Fischer et al. 2006; Thomsen et al. 2002).

Exposure to POPs is also a matter of exposure to their metabolites. Experimentally it has been shown that PBDEs are transformed into hydroxylated metabolites (OH-PBDEs) (Hakk and Letcher 2003) via routes similar to those for PCB congeners (Letcher et al. 2000). The metabolism of BDE-47 in mice and rats (Örn and Klasson Wehler 1998) generates tri- and tetrabrominated hydroxylated metabolites, of which six OH-tetraBDEs and three OH-triBDEs have been identified structurally in rats (Marsh et al. 2006). BDE-99 has been found to be metabolized to two pentabrominated and two tetra-brominated hydroxylated diphenyl ethers in the rat (Hakk et al. 2002), and BDE-100 has generated five OH-pentaBDEs and six OH-tetraBDEs in the rat (Hakk et al. 2006). A mixture of seven environmentally relevant PBDEs has been shown to form up to 16 OH-PBDEs that are retained in rat blood (Malmberg et al. 2005). Hydroxylated metabolites of PBDEs have also been reported to be retained in mouse plasma after exposure to a commercial pentaBDE product (Qiu et al. 2007). OH-PBDEs have been identified as metabolites of BDEs 47, 99, 100, and 153 in urine and feces from female mice (Chen et al. 2006; Staskal et al. 2006). It is likely that OH-PBDEs, which have structural similarities with thyroid hormones, also are retained in human blood.

Hydroxylated PBDEs substituted with the hydroxyl group in an *ortho* position to the ether bridge appear to be primarily of natural origin (Malmvärn et al. 2005; Marsh et al. 2004), and have been identified structurally in biota from marine environments. There are thus two sources of OH-PBDEs in the environment: natural production in marine ecosystems, and anthropogenic formation via the metabolism of PBDEs.

The aim of the present study was to assess human exposure to PBDEs in Nicaragua and, if high concentrations were detected, also to search for OH-PBDE metabolites in their blood. The study groups included potentially highly exposed children working as scrap scavengers at a large municipal waste disposal site. Also, we investigated women from the urban and rural areas of Managua with varying consumption of potentially contaminated fish from the nearby lake. The study, which was performed in 2002, was part of a program investigating exposure to organohalogens (Cuadra et al. 2006), heavy metals (Cuadra 2005), and respiratory irritants, as well as work-related injuries and respiratory disease in child workers at the waste disposal site.

## Material and Methods

### Setting

**Figure 1:**
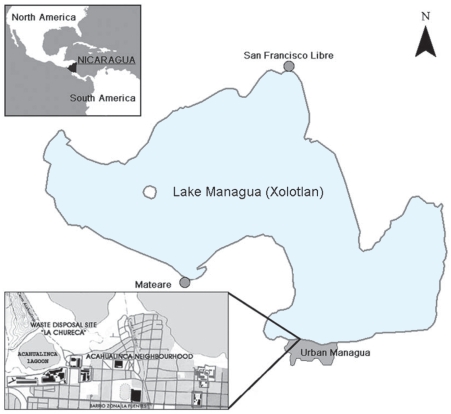
Map of the Managua area, Nicaragua. The locations of Mateare, San Francisco Libre, urban Managua, and the waste disposal site “La Chureca” are shown.

Managua, the capital of Nicaragua, is situated on the shore of Lake Managua (Figure 1). The lake, the second largest in Nicaragua, serves as the recipient of domestic and industrial wastewater from the city, and receives the superficial run-off from its drainage basin, which is intensively cultivated. Fish from the lake are an important part of the diet, not only for the population living in the rural fishing villages, but also for parts of the Managuan urban population.

The main municipal domestic and industrial waste disposal site in Managua, La Chureca, which covers an area of 7 km^2^, is located directly on the south shore of the lake. Approximately 1,000 persons work regularly at the waste disposal site, collecting recyclable waste for selling. More than 50% of those workers are children < 18 years of age. A thick cloud of smoke covers the area as the waste is burned to retrieve iron and other materials. Electronic waste is rarely found at the dump site. The waste is not compressed, the sunlight is intense, and a constant breeze from the lake sweeps the area. Thus, substantial amounts of airborne dust are generated.

### Study groups

The ethics committees at Lund University and The National Autonomous University of Nicaragua–Managua (UNAN–Managua) approved the study protocol, and written informed consent was obtained from the participants, and, for children, guardians.

All children currently working at the waste disposal site were identified with the help of a local nongovernmental organization, Centro Dos Generaciones, which maintains an updated register of child workers and their families within the framework of a special program to prevent and eradicate child labor, which has existed for many years. The study of organohalogen exposure was restricted to those in the age range of 11–15 years, in all 64 children working at the waste disposal site and living there or in a nearby area, Acahualinca. With the help of the local school, we selected 80 referent children from Acahualinca. We also recruited a remote reference group, 18 children living 10–20 km away from the waste disposal site in the south and central areas of Managua, by help of the Chateles project, a local nongovernmental organization. None of the referent children had a history of current or previous work at the waste disposal site. All children included shared the same poor socioeconomic conditions. The distribution of age and sex was also similar in the different groups. There was a participation rate of 90% for those working at the waste disposal site, 70% for referents living nearby, and 100% for the remote reference group. The blood sampling took place in May and June 2002.

Detailed information on work history and dietary habits, especially fish consumption, was obtained by a structured interview. Blood was drawn from the cubital vein into evacuated plain tubes (Vacutainer, Rutherford, NJ, USA) and then centrifuged. The serum was transferred to acetone-washed glass bottles, frozen, and kept at –20°C until it was chemically analyzed in Stockholm, Sweden.

According to criteria described below, children were selected and stratified in terms of their work experience at the waste disposal site, the area where they were living, and their fish consumption. Five distinct groups for pooling of serum were identified (Table 1): Pool 1: children living at the waste disposal site, who had worked there for 4–10 years (median, 6 years); half of the children had been living at the dump all their life, the other half for 5–11 years; pool 2: children living in the nearby area, Acahualinca, who had worked at the waste disposal site for 4–12 years or more (median, 6 years); pools 3 and 4: children living in Acahualinca but not working at the dump; pool 5: children living in a remote urban area.

Women with markedly different patterns of fish consumption, residing in poor urban and rural districts in the Managua area, were also included in the study with the help of local authorities, local health centers, and fishermen’s cooperatives—in all, 32 women. There were sufficient quantities of serum to assemble another four distinct pools for analysis: pool A: women 15–17 years of age, living in fishermen’s families in San Francisco Libre, a fishing village on the rural northeast side of the lake; pool B: women 20–29 years of age living in fishermen’s families in Mateare, another fishing village 25 km from urban Managua; pool C: women from urban Managua 18–25 years of age; pool D: women from urban Managua 42–44 years of age. The urban women consumed either no fish or only a negligible amount. All the subjects lived under similar underprivileged socioeconomic conditions. Demographic data are presented in Table 1. The blood sampling was performed in July 2002.

The locations of the study areas are shown in Figure 1. The areas selected for the child referents as well as for the urban and rural women are characterized by poverty, and are similar in the degree of access to the health and education systems [Instituto nacional de estadisticas y cencos (INEC) 2005].

The individual serum samples obtained were pooled within each given group. We also had access to already assembled pools of serum from a previous study (Cuadra et al. 2006). These “new” and “old” pools included the same set of children, with the exception of 1–3 individuals in each pool for whom enough quantities of serum were lacking to include in the new pool (Table 1). For the child workers and the referents, the PBDE analyses were performed in duplicate pooled samples. For the PBDE metabolites, there was not sufficient serum for duplicate analyses to be carried out for the children, except for one pool. For the young women new pools were assembled, only one of which was large enough to permit duplicate analyses to be performed.

### Chemicals

The reference and study compounds are presented with their numbers in accordance with Ballschmiter et al. (1993). The PBDEs were synthesized as previously described (Marsh et al. 1999; Örn et al. 1996), except for the decaBDE that was commercially available (Fluka Chemie, Buchs, Switzerland). The hydroxylated polybrominated diphenyl ethers were assessed as their methoxylated derivatives for the identification and quantification of any OH-PBDEs. The methoxylated (MeO)-PBDEs were also synthesized in-house (Marsh et al. 2003).

Diazomethane, used for methylation of OH-PBDEs, was prepared from *N*-methyl-*N*-nitroso-*p*-toluenesulfonamide (Diazald) obtained from Sigma-Aldrich (Steinheim, Germany) and synthesized in house (Fieser and Fieser 1967).

### Instruments

The PBDE and OH-PBDE analysis was performed by gas chromatography/mass spectrometry (GC/MS) using the instrumental set up described elsewhere (Fängström et al. 2005). Automated 1-μL injections to the GC were made on a septum-equipped temperature programmable injector fitted with a high performance insert connected directly to a DB-5 HT capillary column (15 m × 0.25 mm i.d., 0.1 μm film thickness; J&W Scientific, Folsom, CA, USA) using helium as the carrier gas at a head pressure of 3 psi. The PBDE and the methylated OH-PBDE congeners were analyzed by selected ion monitoring by scanning for the negative bromide *m/z* 79 and 81 ions (Buser 1986) formed by electron capture reactions at chemical ionization (ECNI) with methane (5.0; AGA, Stockholm, Sweden) as the electron thermalization buffer gas at 5.6 torr and a primary electron energy level of 70 eV. All chromatographic data were collected, analyzed, and quantified using the proprietary ICIS2 software from Thermofinnigan (Bremen, Germany). The linear relationship of the GC/MS system was determined and the quantifications were performed using a single-point external standard within the concentration range of the linear relationship.

### Extraction and cleanup

The general method used for extraction is described elsewhere (Hovander et al. 2000), but it was modified slightly through replacing *n*-hexane by cyclohexane. The substitution of cyclohexane for *n*-hexane was done after completion of a recovery study of the analytes, which showed the results to be similar to those with use of *n*-hexane.

In brief, 5 g of serum was weighed into a test tube. Two internal standards (BDE-77, 0.3 ng/sample; and 6-OH-BDE-85, 0.5 ng/sample) were added, vortexed and left to equilibrate overnight in a refrigerator before extraction. Hydrochloric acid and 2-propanol were added to denaturize the proteins and release the lipids and the organohalogen compounds. The analytes were extracted by cyclohexane and methyl *tert*-butyl ether (1:1). The organic phase was washed with a solution of potassium chloride (1%). The lipids were determined gravimetrically by constant weight determination.

#### Separation of neutral and phenolic compounds

The analytes and lipids were dissolved in cyclohexane. Potassium hydroxide (0.5 M) was added for partitioning the neutral and acidic analytes. The isolated alkaline solution was acidified with hydrochloric acid and the protonated phenols were extracted with cyclohexane:methyl *tert*-butyl ether (9:1). The solvent volume was reduced to approximately 0.5 mL before derivatization of the halogenated phenols with diazomethane.

#### Derivatization-rate test

Seven OH-PBDEs were used for determining the rate of methylation. Diazomethane was added to five test tubes containing exactly the same amount of each of the seven compounds. Methylation was interrupted after 30 min, 1 hr, 3 hr, and 5 hr, the additional test tube being left overnight. The methylation was stopped by reducing the excess of diazomethane under a gentle stream of nitrogen. An injection standard was added before analysis on GC/MS (ECNI). It was shown that 30 min was sufficient for quantitative methylation.

#### Lipid removal

The neutral compounds and the methoxylated derivatives of the phenolic compounds were isolated free from the lipids after the samples, containing the neutral and the methylated phenols dissolved in cyclohexane, had been treated with concentrated sulfuric acid.

#### Silica/sulfuric acid clean up

Pasteur pipette columns were packed with silica gel (0.1 g) and silica/sulfuric acid gel (2:1; 1 g) for cleanup of both the neutral and phenolic fraction. The columns were pre-washed and the analytes similarly eluted with cyclohexane:DCM (dichloromethane) (1:1, 6 mL) and DCM (10 mL).

#### Silica column

Because the sample volumes were significantly reduced (50 μL–200 μL) before GC/MS analysis, an additional silica column was used to remove any additional impurities in the neutral and the methylated phenol fractions. Two fractions were collected from the silica column: a first fraction in cyclohexane (3 mL) and a second fraction in DCM (6 mL). In both cases, fraction 2 contained the compounds of interest. The solvent volume was reduced to dryness and the residue was dissolved in *n*-hexane (200 μL). An injection standard (100 μL) was added to each sample: 4′-MeO-BDE-121 (0.8 ng/sample) to the neutral analyte fraction, and BDE-66 (0.5 ng/sample) to the phenol analyte fraction.

### Quality control of the analysis

The workup of all samples was performed in a clean room. The solvents and the dilutions employed were checked for any potential contamination of the PBDE analytes before their use. Blanks were run and checked before any samples were treated. The samples were analyzed in duplicates using procedure blanks. Instrumental limits of detection (ILOD) were provided by use of corresponding reference compounds and were defined as three times the signal-to-noise ratio (S/N). The limit of quantification (LOQ) was calculated as three times the ILOD amount for each compound (Tables 2 and 3), by approximation of the sample volume to 100 μL and of the lipids to be 20 mg extracted from 5 g plasma.

## Results

**Figure 2:**
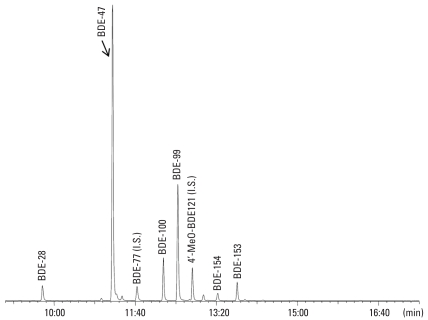
GC/MS (ECNI) chromatogram of PBDEs in serum from children working and living on a waste disposal site in Managua, Nicaragua. I.S., internal standard. The identified PBDE congeners are shown by their abbreviated compound numbers. The instrument was set to trace bromide ions (*m/z* 79/81).

The abundance of the PBDE congeners can be seen in a GC/MS (ECNI) chromatogram of bromide ions (*m/z:* 79, 81) from the neutral fraction in serum from children who were living and working at the waste disposal site (Figure 2). Even though the analysis did not discriminate for any PBDE congeners, only 10 were quantifiable (Table 2). The results for duplicate samples were generally in close agreement with each other, the differences between samples involving slightly differing pool compositions (a versus b in Tables 1–3) being somewhat greater.

BDE-47 was the dominant PBDE congener in the pools, followed by BDE-99 at about half or less the concentration of BDE-47. BDE-100 and BDE-153 were the third and fourth most abundant PBDE congeners, respectively. BDE-209 was one of the least abundant congeners, but still quantitated in all pools. The levels of BDE-17 and BDE-128 were below LOQ [< 0.60 and < 0.80 pmol/g lipid weight (l.w.) respectively] in all of the pools. Except for BDE-203, which was below LOQ (< 1 pmol/g l.w.), octa- and nonaBDEs were not detected (Figure 2).

The children working and living at the city dump (pools 1a and 1b) had the highest levels of PBDEs, approximately 20–50 times as high as those of the referent children in urban Managua (pools 3, 4, and 5) when one considers PBDEs with up to six bromine atoms. In contrast, the difference between the groups was minimal for BDE-183 and BDE-209 (Table 2).

In the urban children who did not work at the waste disposal site, the levels of the sum of the PBDEs as well as of several individual congeners were somewhat higher in those eating fish from the lake than in those who did not eat fish. However, the urban women with little or no fish consumption clearly had higher PBDE levels than the women living in rural fishing villages in the Managua area, who consumed large amounts of fish from the lake.

**Figure 3:**
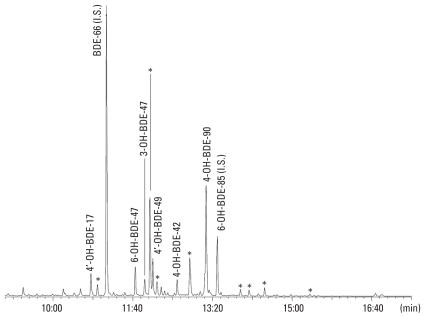
GC/MS (ECNI) chromatogram of phenolic polybrominated diphenyl ethers (OH-PBDEs) in serum from children working and living on a waste disposal site in Managua, Nicaragua. I.S., internal standard. The identified and the unidentified OH-PBDEs are shown by compound abbreviations and asterisks, respectively. The instrument was set to trace bromide ions (*m/z* 79/81).

The OH-PBDEs were analyzed as methyl derivates (MeO-PBDEs). A chromatogram of methyl ethers of the OH-PBDEs in the children working and living at the dump is shown in Figure 3, confirming the presence of 19 OH-PBDEs in human plasma. Six of the OH-PBDEs were tentatively identified in terms of their retention times compared with those of authentic reference compounds that were available, and in terms of their signal for *m/z* = 79/81 in bromide ion trace analysis (ECNI). The chromatographic peak patterns were also compared with those found in a chromatogram, analyzed in parallel, of a plasma sample obtained from rats that had been exposed to a mixture of seven equimolar PBDE congeners (BDEs 47, 99, 100, 153, 154, 183, and 209) (Malmberg et al. 2005). The position of the hydroxyl group could be identified in 17 mono-hydroxylated PBDEs (10 *meta*, 6 *para*, and 1 *ortho* hydroxylated PBDEs) according to OH-PBDE mass fragmentation (electron ion-ization) (Athanasiadou et al. 2006). The six OH-PBDEs that were tentatively identified were quantified (Table 3). Results for duplicate samples were generally in close agreement. Nine OH-PBDEs [each marked with an asterisk (*) in Figure 3] were identified as being the same metabolites that were formed in the experimental study where rats were exposed to an artificial PBDE mixture (Malmberg et al. 2005), but not yet structurally identified due to a lack of appropriate standards.

Of the metabolites having three to four bromines, it appeared that 4-OH-BDE-17 and 4′-OH-BDE-49 were the dominant phenolic metabolites. The peak marked 4-OH-BDE-90 is a double peak determined by two pentabrominated hydroxylated diphenyl ethers. The peak was quantified relative to the 4-OH-BDE-90 standard.

The pools obtained from children living and working at the dump showed a markedly higher serum level of hydroxylated metabolites than found in any of the other pools (Table 3).

## Discussion

The occupational exposure to PBDEs in children working at a waste disposal site, and a substantial background level of exposure to PBDEs in the urban population, are clearly shown. The PBDE concentrations in the serum of children working and living at the waste disposal site are among the highest ever reported. Most interesting, we found that hydroxylated PBDE metabolites are retained in human serum, just like OH-PCBs.

### Design considerations

We used pooled samples to keep the amount of blood sampled as low as possible in this population of mal-nourished children but also for economic reasons not allowing us to analyze individual samples for PBDEs. The result from a pooled sample is equivalent to the mean for the group in question, but its weakness is that there is no information concerning the variation between individuals. To partially overcome this limitation, we selected the subjects to be included very carefully, so that the separate pools would clearly differ with regard to the factors we wished to investigate (the effects of work at the waste disposal site, the area of living involved, and fish consumption), but as homogeneous as possible within each pool.

The child workers at the waste disposal site who were enrolled represent most of the child workers of their age. As referents, we enrolled children not involved in scrap scavenging, but living in the same area, and children from low-income families living far away from the waste disposal site. The body mass index, blood iron content, and blood lipid concentration found in the child workers and the referents were comparable (Cuadra 2005). A previous study of 103 scrap scavengers and 103 referent children from Acahualinca 6–15 years of age (the children selected in our study being a subgroup of these children) had indicated similar prevalence of chronic malnutrition (height for age below the 50th percentile, obtained from Epi-Info: Nut-stat 2nd revision; Centers for Disease Control and Prevention 2007) and acute malnutrition (weight per age below the 50th percentile, obtained from Epi-Info: Nut-stat 2nd revision) in child workers and referents—around 85% prevalence for chronic and around 65% for acute malnutrition (Hernandez Romero D, personal communication). Thus, there are indications that the desired socioeconomic similarity of the groups, including nutritional status, was achieved.

### Exposure to polybrominated diphenyl ethers

**Figure 4:**
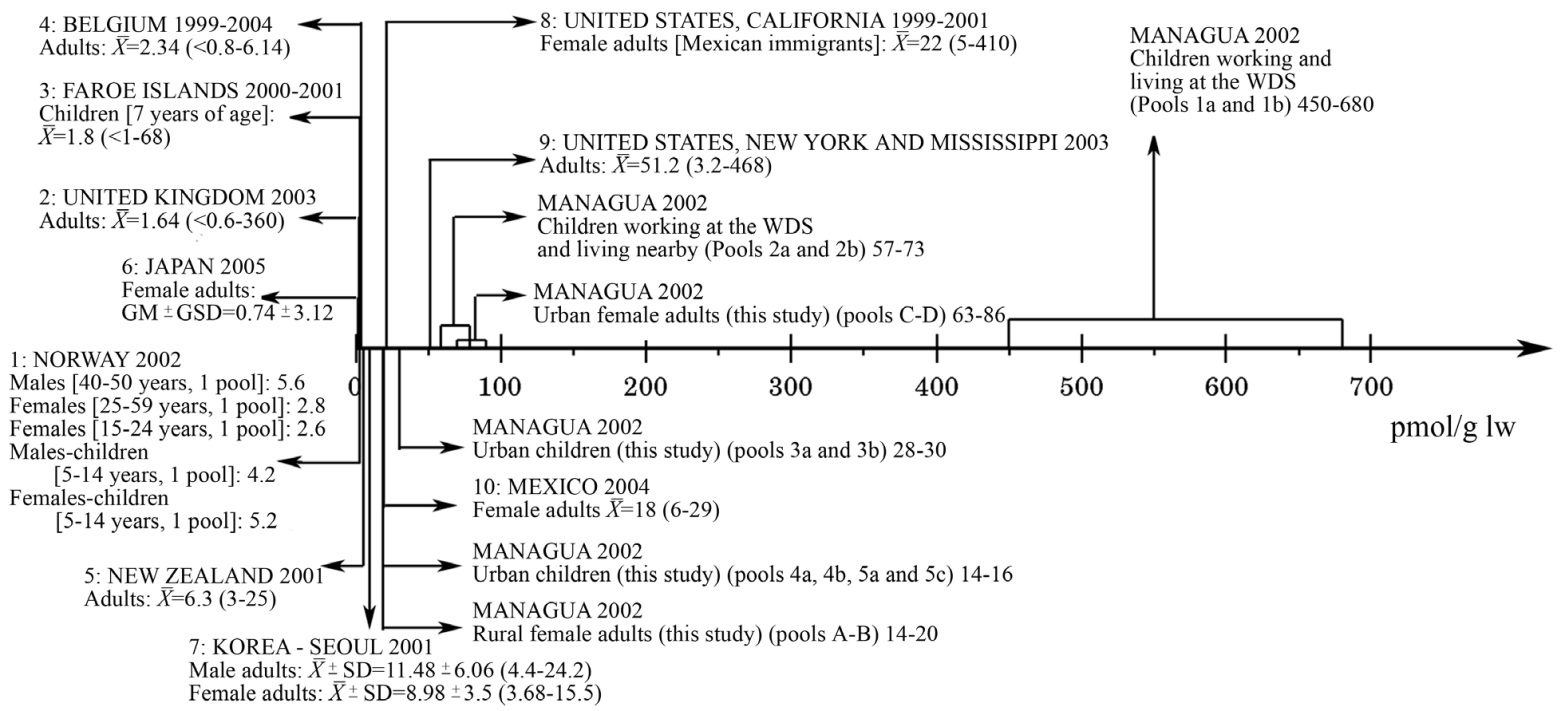
Serum levels of BDE-47 (pmol/g l.w.) in different regions. Data are from: 1: Thomsen et al. (2007); 2: Thomas et al. (2006); 3: Fängström et al. (2005); 4: Covaci and Voorspoels (2005); 5: Harrad and Porter (2007); 6: Inoue et al. (2006); 7: Kim et al. (2005); 8: Bradman et al. (2007); 9: Schecter et al. (2005b); 10: Lopez et al. (2004). For comparison, reported concentrations were transformed into serum concentrations in pmol/g l.w. when needed. &*Xmacr*; = mean; X̃= median; GM, geometric mean; GSD, standard deviation of GM; WDS, waste disposal site.

**Figure 5:**
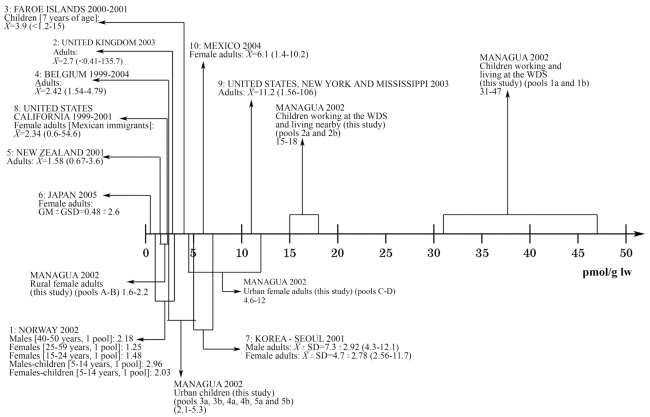
Serum levels of BDE-153 (pmol/g l.w.) in different regions. Data are from: 1: Thomsen et al. (2007); 2: Thomas et al. (2006); 3: Fängström et al. (2005); 4: Covaci and Voorspoels (2005); 5: Harrad and Porter (2007); 6: Inoue et al. (2006); 7: Kim et al. (2005); 8: Bradman et al. (2007); 9: Schecter et al. (2005b); 10: Lopez et al. (2004). For comparison, reported concentrations were transformed into serum concentrations in pmol/g l.w. when needed. X̄= mean; &*X*tilde; = median; GM, geometric mean; GSD, standard deviation of GM; WDS, waste disposal site.

**Figure 6:**
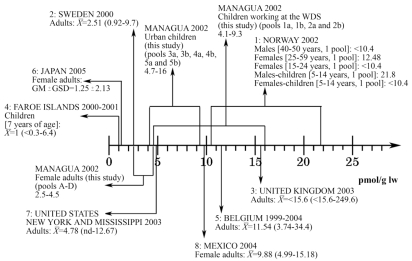
Serum levels of BDE-209 (pmol/g l.w.) in different regions. Data are from: 1: Thomsen et al. (2007); 2: Thuresson et al. (2005); 3: Thomas et al. (2006); 4: Fängström et al. (2005); 5: Covaci and Voorspoels (2005); 6: Inoue et al. (2006); 7: Schecter et al. (2005b); 8: Lopez et al. (2004). For comparison, reported concentrations were transformed into serum concentration in pmol/g l.w. when needed. X̄= mean; &*X*tilde; = median; GM, geometric mean; GSD, standard deviation of GM; nd, nondetectable; WDS, waste disposal site.

The present data from Nicaragua should be put in context of PBDE exposures elsewhere, as illustrated in Figures 4–6, in which observations from different areas worldwide are presented. Unexpectedly, the children living and working at the waste disposal site had very high levels of medium BDEs, with the group mean being almost an order of magnitude higher than hitherto reported. In all the pools, BDE-47 was the dominant PBDE congener, followed by BDE-99, BDE-100, and BDE-153. Thus, the congener profile suggests exposure mainly to the technical pentaBDE product (La Guardia et al. 2006). This is in accordance with the absence of electronic waste at the waste disposal site.

Also, quite unexpectedly, the levels of the medium BDEs observed among referent children and young and middle-age women living in an unindustrialized urban area in the second poorest country in the Americas were comparable to contemporary observations in the United States (Bradman et al. 2007; Fischer et al. 2006; Hites 2004; Lunder and Sharp 2004; Morland et al. 2005; Schecter et al. 2005b; She et al. 2007), and much higher than in industrialized countries in Europe and Asia (Figures 4 and 5). BDE-209 was also found in all the pools, with levels as high as reported elsewhere, or even higher (Figure 6).

Many of the children working and living at the waste disposal site (4 of 8 in pool 1a; 6 of 11 in pool 1b) had been living there all their life. Thus, both pre- and postnatal PBDE exposure is likely to have occurred. This could tentatively contribute to their present levels, when one takes the presumably long half-life (several years) of medium BDEs into account (Geyer et al. 2004).

In contrast, the level of BDE-209 reflects recent exposure only, because the congener has a short half-life in serum, approximately 15 days (Thuresson et al. 2006). The BDE-209 concentrations were rather homogeneous between the different pools (Table 2). A similar pattern was also indicated for BDE-183, which has an apparent half-life of approximately 3 months.

Our data clearly support the hypothesis that human exposure to PBDE is strongly influenced by dust inhalation and ingestion, rather than by contamination of food. At the waste disposal site, high levels of dust are generated as the waste is burned. In 2005, the mean levels of particulate matter (PM <2.5 μm aerodynamic diameter) at the waste disposal site were 700 μg/m^3^, compared with 100 μg/m^3^ in Acahualinca nearby (Hernandez Romero D, personal communication). We hypothesize that the uncontrolled burning of waste also plays a major role as a source of PBDE exposure in the general population. La Chureca is the major waste disposal site in Managua, but due to deficiencies in the local waste management system, many informal small open dumps where wastes are burned are found in the city, and daily backyard burning is common. This also happens in the rural areas, but the burden of waste is far less than in the urban areas. Thus, the situation for PBDEs may be similar to that of dioxins, with uncontrolled burning of waste being a major source of exposure (Hedman et al. 2005).

Elevated levels of PBDEs in soil due to open burning of electronic waste has been reported from China (Leung et al. 2007), and PBDEs have been found in leachates from open waste disposal sites in Canada (Danon-Schaffer et al. 2006) and in Japan (Osako et al. 2004). Also, elevated levels of PBDEs have been reported in breast milk from women living near an open waste site in India (Kunisue et al. 2006).

That the findings for the women differ markedly in their fish consumption indicates that factors linked to urban dwelling were more important for the levels of medium brominated PBDEs than high fish consumption (Table 2, pools A and B vs. pools C and D). We have no detailed dietary information in these subjects, but it is unlikely that their diet, based on rice and beans, differs markedly, except that those in the fishing villages eat more fish and accordingly less meat. Such a marked urban–rural gradient has not been reported previously. In contrast, the levels of PCBs were higher in the women from the rural areas than in the urban women, indicating the importance of dietary exposure through fish consumption (Table 1). A similar pattern was also observed for 4,4′-dichlorodiphenyldichloroethylene (Cuadra et al. 2006). It is notable that the concentrations of both BDE-47 and BDE-99 (~ 600 and 300 pmol/g l.w., respectively) were higher than the level of the dominant PCB congener, CB-153 (195 pmol/g l.w.) in the children working and living at the waste disposal site. For the other children, the CB-153 levels were higher by a factor of approximately 10 on a molar level.

Our data clearly indicate that exposure to PBDEs through inhalation is significant in this population, and of greater magnitude than dietary exposure. However, we observed somewhat higher PBDE levels among the nonworking children who ate fish from the lake (pool 3) than in nonconsumers of fish (pools 4 and 5). Thus, fish from the lake may also be a source of exposure to medium BDEs. This is in line with previous findings of a correlation between fish consumption and the level of BDE-47 (Ohta et al. 2002; Sjödin et al. 2000).

### PBDE metabolites

Selected OH-PBDE metabolites were detected and quantified in serum, indicating their active retention in blood. This is a behavior that these particular OH-PBDEs share with a limited number of structurally defined OH-PCBs (Letcher et al. 2000; Malmberg et al. 2005). This result supports our hypothesis that OH-PBDEs, fulfilling certain structural criteria, do accumulate in humans in a manner similar to certain OH-PCBs.

We tentatively identified and quantified six OH-PBDE congeners in the children working and living at the waste disposal site. However, there is a lack of hydroxylated PBDE reference standards having five and more bromines. This limitation in available reference substances is a major obstacle for identification of OH-PBDEs, because co-elution of methylated OH-PBDEs is to be expected. However, we could compare the present OH-PBDE profile with the profile obtained in an experimental study of rats given equimolar doses of the major PBDEs (Malmberg et al. 2005). At least six of the OH-PBDEs in the previous rat study were also formed and retained in the serum from the child workers (Figure 3).

For four of the OH-PBDEs that were identified, the hydroxyl group was substituted in a *para* position, and for one in a *meta* position. One OH-PBDE was substituted in an *ortho* position. These results indicate formation from BDE-47 metabolism also in humans (cf. studies in the rat), and not from natural (dietary) origin (Malmvärn et al. 2005; Marsh et al. 2004). The pentabrominated diphenyl ethers (BDE-85, BDE-99, and BDE-100), which were prevalent in the children (Figure 2, Table 2), form OH-PBDE metabolites (Hakk et al. 2002, 2006) as shown in Figure 3. However, OH-PBDEs with six to seven bromine atoms were also indicated.

### Multiple exposures and risk

It is not possible yet to adequately discuss the full implications of human exposure to either PBDE compounds or their OH-PBDE metabolites from a toxicologic perspective. But while awaiting the toxicologic data, at least exposure assessment can be performed.

We found not only exposure to PBDE and its metabolites but also elevated levels of heavy metals, organochlorine pesticides and PCBs in these children (Cuadra 2005; Cuadra et al. 2006). The need for an integrated risk evaluation is evident when considering the fact that several of the substances have a common critical organ—the developing brain. The levels of exposure to xenobiotics that we have observed are directly relevant to risk assessment with regard to reproductive outcomes. Many of the teenage girls will be mothers in the near future. As many as 21% of the adolescent females in Managua were either pregnant or already mothers in 2001 (INEC 2005). Also, the need to investigate the toxicologic effect of chemical mixtures in connection with malnutrition has been taken up recently. In our population this is clearly relevant, because malnutrition was prevalent.

### Conclusions

We studied a vulnerable population of children and adolescents living under extreme socioeconomic conditions, in which exposure to multiple hazardous chemicals is evident. The data collected clearly indicate that dust is a significant source of exposure to PBDEs in this population. The unexpected finding of high levels of PBDEs and their OH-PBDE metabolites in urban children in a developing country highlights the need for worldwide exposure assessment of emerging pollutants, not just in the developed countries. This is not only of scientific interest, but indeed a matter of concern for society.

## Correction

In the original manuscript published online, BDE-153 and BDE-154 were reversed in position in Figure 2; they have been corrected here.

## Figures and Tables

**Table 1:** Sociodemographic characteristics for children working at a waste disposal site (WDS) in Managua (pools 1–2) and referents (pools 3–5), and for females with varying consumption of fish from Lake Managua (pools A–D).

Group	No. (CAS)	Sex: no. [M (F)]	Age^a^ (years)	Fish consumption (meals/month)^a^	Domicile: years duration in dwelling^a^	Location	Work at WDS: age of onset^a^	Work at WDS: years worked^a^	Work at WDS: hours/day^a^	Work at WDS: days/week^a^	∑PCBs (ng/g l.w.)
Pool 1a^b^	8 (7)	4 (4)	14 (13–15)	1.5 (0–8)	12 (5–16)	WDS	7.5 (6–10)	6 (4–10)	3.5 (2–12)	5.5 (2–7)	—
Pool 1b^c^	11 (8)	6 (5)	14 (13–15)	2 (0–8)	13 (5–16)	WDS	7 (6–10)	6 (4–10)	4 (2–12)	6 (2–7)	540
Pool 2a^b^	21 (15)	15 (6)	14 (12–15)	2 (0–8)	13 (7–15)	Acahualinca	8 (2–10)	6 (4–12)	5 (3–12)	3 (1–7)	—
Pool 2b^c^	23 (17)	16 (7)	14 (12–15)	2 (0–8)	13 (7–15)	Acahualinca	8 (2–11)	6 (4–12)	5 (3–12)	3 (1–7)	530
Pool 3a^b^	15 (15)	5 (10)	14 (13–15)	2 (2–8)	13 (6–15)	Acahualinca	—	—	—	—	—
Pool 3b^c^	16 (16)	6 (10)	14 (11–15)	2 (2–8)	13 (6–15)	Acahualinca	—	—	—	—	390
Pool 4a^b^	8 (8)	3 (5)	13.5 (13–15)	0	13 (6–14)	Acahualinca	—	—	—	—	—
Pool 4b^c^	10 (10)	5 (5)	14 (13–15)	0	13 (6–14)	Acahualinca	—	—	—	—	230
Pool 5a^b^	8 (8)	4 (4)	13.5 (12–14)	0	11.5 (10–14)	Urban Managua	—	—	—	—	—
Pool 5b^c^	11 (11)	5 (6)	13 (12–14)	0	12 (10–14)	Urban Managua	—	—	—	—	160
Pool A	5	(5)	15–17	4 (4–8)	8 (7–17)	Fishing village	—	—	—	—	286
Pool B	3	(3)	20–20	8 (8–16)	20	Fishing village	—	—	—	—	476
Pool C	3	(3)	18–25	0 (0–0)	10 (9–11)	Urban Managua	—	—	—	—	176
Pool D	4	(4)	42–44	0 (0–4)	12.5 (10–17)	Urban Managua	—	—	—	—	—

Abbreviations: CAS, currently attending school; F, female; M, male. Serum levels of PCBs are also given [data from present study and Cuadra et al. (2006)].

a Median (range).

b Samples with serum enough for duplicate analyses; all subjects also included in pool b.

c Pool b used by Cuadra et al. (2006).

**Table 2:** Concentrations (pmol/g l.w.) of individual PBDE congeners (molecular weight) in children working at a waste disposal site in Managua (pools 1–2) and referents (pools 3–5), and for females with varying consumption of fish from Lake Managua (pools A–D).

	Lipid (%)	BDE-28 (407)	BDE-47 (486)	BDE-66 (486)	BDE-100 (565)	BDE-99 (565)	BDE-85 (565)	BDE-154 (644)	BDE-153 (644)	BDE-183 (722)	BDE-209 (959)	∑PBDE^a^
Pool 1a^b^	0.41/0.41	24/22	680/600	12/11	111/108	309/308	35/35	22/20	47/45	2.4/2.5	4.3/6.3	1,250/1,160
Pool 1b^c^	0.43	16	450	7.9	71	210	20	18	31	2.3	9.3	840
Pool 2a^b^	0.38/0.37	2.4/1.7	73/66	0.66/0.66	17/19	19/20	1.8/2.2	5.1/10	18/18	2.4/2.3	3.1/4.1	144/145
Pool 2b^c^	0.40	1.7	57	0.51	16	18	1.8	9.2	15	1.7	3.8	125
Pool 3a^b^	0.39/0.41	1.4/1.1	30/28	0.63/0.61	8.4/6.2	12/8.9	1.1/0.69	4.3/3.0	5.3/3.7	1.1/1.1	6.2/5.2	72/60
Pool 3b^c^	0.41	0.87	29	0.66	6.6	9.5	1.1	2.8	4.5	1.3	10	66
Pool 4a^b^	0.44/0.42	0.6/0.6	11/11	0.29/0.30	2.0/2.1	4.2/5.0	0.33/0.26	1.8/3.5	2.1/2.3	1.3/3.9	4.7/11	30/42
Pool 4b^c^	0.41	0.73	16	0.49	3.7	5.9	0.56	2.2	3.2	2.5	13	48
Pool 5a^b^	0.41/0.41	0.8/0.8	14/15	0.40/0.41	3.3/3.6	6.1/6.9	0.51/0.52	2.9/3.1	2.6/2.7	0.94/1.0	7.0/5.0	40/40
Pool 5b^c^	0.22	0.69	14	0.49	3.1	6.0	0.56	2.2	2.5	1.9	16	47
Pool A	0.35	0.83	20	0.47	4.2	11	0.70	2.0	2.2	1.8	4.5	49
Pool B	0.51	0.38	14	< LOQ	3.3	3.7	0.28	1.8	1.6	< LOQ	3.7	31
Pool C	0.41	3.6	86	0.67	16	14	1.7	2.0	12	0.79	4.1	142
Pool D	0.71/0.66	1.9/1.9	63/73	1.1/1.3	10/12	25/29	2.1/3.3	2.5/3.1	4.6/6.2	0.39/0.58	2.5/3.5	114/135
ILOD^d^		15	12	15	15	18	15	20	38	40	180	
LOQ^e^		0.56	0.36	0.46	0.40	0.48	0.40	0.46	0.88	0.84	2.8	

Weight-based concentrations can be calculated by multiplying with the molecular weight given in the table. The lipid content is given for the pools to allow for fresh weight calculations. Dual values are results of duplicate analyses.

a Sum of the individual PBDE congeners presented in the table.

b Samples with serum enough for duplicate analyses; all subjects also included in pool b.

c Pool b as in Cuadra et al. (2006).

d ILOD provided with corresponding reference compound 3 times the signal-to-noise ratio expressed in femtograms (fg).

e A concentration of 3 times ILOD in a total sample volume of 100 μL (prior analysis) and 20 mg lipids (pmol/g l.w.).

**Table 3:** Concentrations pmol/g l.w.) of methyl derivatives of individual OH-PBDEs (molecular weight) (in children working at a waste disposal site (WDS) in Managua (pools 1–2) and referents (pools 3–5), and for females with varying consumption of fish from Lake Managua (pools A–D).

	Lipids (%)	4′-OH-BDE-17 (438)	6-OH-BDE-47 (516)	3-OH-BDE-47 (516)	4′-OH-BDE-49 (516)	4-OH-BDE-42 (516)	4-OH-BDE-90 (594)	∑OH-PBDEs^a^
Pool 1a^b^	0.41/0.41	17/16	12/13	6.5/4.5	18/14	9.6/6.4	54/45	120/100
Pool 1b^c^	0.43	2.5	11	2.2	4.4	2.8	16	42
Pool 2a^b^	0.38	1.3	4.7	0.83	0.56	0.56	0.48	11
Pool 2b^c^	0.40	0.58	4.5	0.33	0.76	0.84	1.1	9.5
Pool 3a^b^	0.39	0.76	2.0	0.32	0.96	0.32	0.70	5.6
Pool 3b^c^	0.41	0.27	2.4	0.21	0.28	< LOQ	0.50	4.1
Pool 4a^b^	0.44	0.44	1.5	< LOQ	0.37	< LOQ	< LOQ	3.1
Pool 4b^c^	0.41	0.31	2.2	< LOQ	0.31	< LOQ	< LOQ	3.7
Pool 5a^b^	0.41	0.55	1.4	< LOQ	0.47	< LOQ	0.27	3.4
Pool 5b^c^	0.22	0.50	1.5	< LOQ	0.53	< LOQ	0.32	3.4
Pool A	0.51	0.36	1.6	< LOQ	0.92	0.31	0.93	5.6
Pool B	0.35	< LOQ	2.5	< LOQ	< LOQ	0.66	0.86	5.4
Pool C	0.41	< LOQ	4.0	1.3	0.79	0.79	0.96	11
Pool D	0.71/0.66	0.97/0.43	3.8/3.7	0.44/0.67	1.3/0.97	0.33/0.42	1.5/1.5	8.7/8.2
ILOD^d^		8	7	7	9	10	7	
LOQ^e^		0.27	0.20	0.20	0.26	0.29	0.18	

Only pool 1a and pool D were analyzed in duplicate.

a Sum of the OH-PBDEs presented in the table.

b All subjects also included in pool b.

c Pool b as in Cuadra et al. 2006.

d ILOD provided with corresponding reference compound 3 times the signal-to-noise ratio expressed in femtogram (fg).

e Limit of quantification, defined as a concentration of 3 times ILOD in a total sample volume (prior analysis) of 100 μL and 20 mg lipids (pmol/g l.w.).
